# Wide Neutrality Window for Block Copolymer Vertical Orientation Using Incongruent Homopolymer Blended Brushes

**DOI:** 10.1021/acsami.5c11923

**Published:** 2025-09-09

**Authors:** Kaitlyn Hillery, Sharif Tasnim Mahmud, Nayanathara Hendeniya, Ava Huth, Caden Chittick, Shaghayegh Abtahi, Boyce S. Chang

**Affiliations:** Department of Materials Science and Engineering, 1177 Iowa State University, Ames, Iowa 50011, United States

**Keywords:** polymer brush, block copolymer, vertical orientation, neutral substrate, incongruent blends, self-assembly, directed self-assembly, responsive surfaces

## Abstract

Distinctive polymer brushes (PBs) play a crucial role in providing a nonpreferential (neutral) surface for vertical orientation of block copolymers (BCPs). This bottom-up approach effectively aligns the formation of vertical lamellar and cylinder lattice structures from the BCP, which is crucial for nanopatterning and other applications. In conventional BCP self-assembly techniques, random copolymer brushes are commonly employed to achieve substrate neutrality. However, these methods face significant drawbacks, including batch variations, synthetic challenges for chemically incompatible monomers, and a narrow neutrality window. Blending homopolymer brushes to achieve mixed chemistry has been demonstrated to be effective for creating neutral substrates but requires precise blend formulations due to the high potential for macrophase segregation. Various approaches have been proposed to mitigate inconsistencies and enhance tunability, albeit with associated costs. Here, we propose a PB system composed of incongruent chain length homopolymers at various blend ratios, which achieves a wide neutrality window for the BCP vertical orientation. These incongruent PB blends demonstrate nonpreferential behavior with lamellar and cylinder forming BCP independent of the brush composition. A canopy effect is proposed as the mechanism that enables the responsive nature of the brush, where a sparse layer of chains will adopt conformations that minimize interfacial energy. By effectively tuning the surface energy of these PB systems, a wide neutrality window is achieved, offering a straightforward, economical template for BCP vertical orientation.

## Graphical abstract

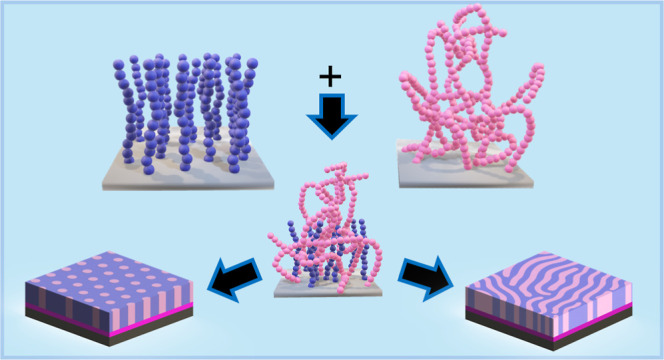

## 1 Introduction

Polymer brushes (PBs) are generally defined as long-chain molecules end-grafted to a substrate or interface and have been extensively investigated for their various applications of surface modification, including colloidal stabilization,^1,2^ lubrication,^3^ quantum dots,^4^ semiconductor fabrication,^5^ and nanostructure templates for microdomains.^6–8^ One of the most important applications of PBs is to facilitate the directed self-assembly (DSA) of block copolymers (BCPs) for semiconductor patterning. BCPs (diblock denoted as A-*b*-B) generally adopt nanoscale lattice structures, including lamellar, cylinders, and gyroids depending on their molecular weight, block composition, and Flory–Huggins interaction parameter (χ).^9^ Through DSA, BCPs can be been applied to create line-space (lamellar) and dot (cylinder) patterns for device fabrication.^10,11^ DSA has been identified as a critical technique for rectifying defects, such as line-edge roughness reduction in EUV lithography. In addition, DSA can be used for density multiplication, where the BCP is guided by using a sparse lithographically defined template with pitch sizes up to 10× larger than the BCP pattern. This effectively enhances the resolution limit without the need for multistep patterning. Here, chemically neutral surfaces are essential to achieve vertical orientation in the “unguided” area between the sparse templates.^6,8,12^

Conventionally, density multiplication utilizes random copolymer (RCP) brushes (also known as statistical copolymer), denoted as poly­(A-*r*-B), for fabricating neutral surfaces that enable vertically oriented (perpendicular) BCP patterns relative to the substrate. The key to vertical orientation is obtaining chemical neutrality on the substrate where a nonpreferential surface does not display bias toward either block of the BCP. In the seminal work by Mansky et al., RCPs were first introduced as a material system for modulating interfacial energy.^13^ This is achieved by synthesizing hydroxyl-terminated poly­(styrene-*r*-methyl methacrylate) (PS-*r*-PMMA) and then thermally grafting these polymers on a silicon oxide (SiO_2_) substrate. Mansky et al. demonstrate that a statistically neutral RCP brush surface was able to promote perpendicular lamellar orientation of PS-*b*-PMMA.^14^ However, RCPs have inherent variations, leading to potential inconsistencies between batches. Additionally, challenges arise in maintaining randomness for high-χ systems due to chemical incompatibility between monomers, resulting in “blocked-like” polymer chains rather than RCPs. These challenges limit the scalability of the RCP process in high-χ systems and present a narrow window of neutrality with inconsistencies between batches of RCPs. In response, other methods of surface modification were developed in an attempt to overcome these challenges, including grafting from side groups, cross-linking mats, and homopolymer blends.^15–18^ Homopolymer blending of PBs is particularly attractive due to a facile and potentially flexible method for achieving a neutral surface.

Homopolymer blends offer an alternative method of neutrality that pivots away from the challenges associated with chemical incompatibility (χ) of the monomers during polymerization for RCPs. The tunable surface chemistry offered by homopolymer blends allows for more control over neutrality by changing the polymer blend composition and chain length. Although homopolymer brushes are used in various applications, few studies have observed the effects of mixing homopolymers in blend brushes for BCP surface energy control. A deterrent to this method is the macrophase separation that can occur with polymer blends, leading to a patchy preferential surface. However, several innovative methods have been employed to mitigate macrophase separation, resulting in a neutral compatible template for BCP self-assembly. One study by Liu et al. successfully developed a two-step grafting method using homopolymers without blending the homopolymers.^19^ This was accomplished by first grafting short-chain PS–OH of lengths of 3, 6, 9, and 20 kg/mol to the substrate, followed by grafting a long-chain 20 kg/mol PMMA–OH through the existing PS brush to the substrate surface. These inserted brushes resulted in a neutral surface that could accommodate a lamellar-forming PS-*b*-PMMA. In a similar study, Liu et al. were also successful in grafting a short-chain congruent homopolymer brush through the addition of a “blending agent” in a one-step method.^20^ These brushes were obtained by first casting a blend solution of 6 kg/mol PS–OH and 6 kg/mol PMMA–OH with a small (5k-*b*-5k) “blender” BCP of PS and PMMA. This “blender” BCP would then be removed along with nongrafted chains after annealing before a larger (52-*b*-52) BCP would be cast and annealed to analyze the surface. This too was successful but could pose challenges in maintaining surface homogeneity when applied to lower molecular weight BCPs, which are required for more aggressive downscaling. More recently, Ceresoli and Sparnacci attempted a one-pot method for homopolymer blend brushes with long-chain PS and PMMA using rapid thermal annealing.^21^ This study grafted long-chain congruent brushes of 16 kg/mol PS and 15 kg/mol PMMA from a blend; after annealing and removing nongrafted chains, a (50-*b*-50) BCP was annealed atop the polymer brush. A narrow window of neutrality was achieved with these one-step casting and rapid annealing techniques. However, large pockets of preferential or non-neutral surfaces were observed outside of these neutral bounds.

The aim of this work is to develop a method for fabricating homopolymer blended brushes that yields a wide range of neutrality for BCP self-assembly. Herein, we discover that incongruent blended brushes provide unprecedented neutrality toward BCPs, obtaining vertical orientation without the strict need of controlling chemical composition. The neutrality of the brush is also demonstrated in solvent-annealed high-χ BCPs. The incongruent brushes demonstrate a highly responsive nature to which we propose a “canopy” theory that aligns with the analytical evidence collected thus far. Collating the effects of polymer chain length, blend composition, and solvent immersion experiments, we attribute the formation of the incongruent brushes to key parameters during brush grafting, including higher dynamic flexibility, enhanced chain mobility, greater segmental freedom of the polymer chains, affinity to the substrate, and mechanochemical exchange.

## 2 Experimental Methods

### 2.1 Materials

Polymers used in these experiments were purchased from Polymer Source Inc., Canada. In short, these will be referred to as their indicated abbreviations displayed in Table 1. Polymers were used as received. As such, the polymers will be referenced with their corresponding abbreviations (e.g., PMMA–OH (α-hydroxypropyl-terminated), Mn = 9.5 kg/mol abbreviated as 10 PMMA, and PS–OH (polystyrene ω-hydroxy-terminated), Mn = 6.0 kg/mol abbreviated as 6 PS. 70% PS–OH and 30% PMMA–OH blend solution abbreviated as 70PS). Clean silicon wafers with a thin native oxide layer were purchased from the university wafers. Toluene and acetonitrile were purchased from Sigma-Aldrich.

**Table 1:** Description of Polymers Used and Abbreviations

polymer	abbr.	PDI	PS cont.: mol % or (kg/mol)	Mn (kg/mol)	abbr. Mn
poly(styrene) ω-hydroxy-terminated	PS–OH	1.05	N/A	6.0	6
		1.09	N/A	10.0	10
				2.7	3
poly(methyl methacrylate), α-hydroxypropyl-terminated	PMMA–OH	1.06	N/A	6.3	6
		1.1	N/A	9.5	10
random, poly(styrene-*co*-methyl methacrylate), α-hydroxyl-ω-tempo moiety terminated	RCP	1.32	54%	7.0	7
		1.4	50%	17.0	17
		1.35	50%	44.0	44
poly(2-vinylpyridine) α-hydroxypropyl-terminated	P2VP–OH	1.05	N/A	9.6	10
poly(styrene-*b*-methyl methacrylate)	BCP	1.09	PS (25)-*b*-PMMA (26)	N/A	25-*b*-26
	BCP	1.06	PS (33)-*b*-PMMA (17)	N/A	33-*b*-17
poly(styrene)-*b*-poly(2-vinyl pyridine)	BCP	1.05	PS (26)-*b*-P2VP (26)	N/A	26-*b*-26

### 2.2 Characterization Methods

Atomic force microscopy (AFM) phase and height maps were obtained using tapping mode with an Asylum Research MFP-3D. Ellipsometry was obtained using J.A. Woollam Alpha-SE and M-2000 for polymer brush thickness. X-ray photoelectron spectroscopy (XPS) VersaProbe was used for the film composition determination. Grazing-incidence small-angle X-ray scattering (GISAXS) (Xenocs Xeuss 2.0) was performed to determine the structural periodicity of the BCP domains. The domain spacing (*L*_0_) of the lamellar features was calculated from the primary, q* scattering peak using eq 1.

1 $$L_{0}=2\pi /q^{*}$$

### 2.3 Sample Preparation

#### 2.3.1 Polymer Brushes

Standard solutions of PS–OH and PMMA–OH and random, PS-*co*-PMMA–OH were prepared in toluene to yield a 1.0% (wt) solution and then filtered using a 0.45 μm syringe filter to remove contaminates. Aliquots of homopolymer standard solutions were mixed to create a 1% in-solution blend of PS and PMMA with varying concentrations of Mn. The blend solutions were mixed via pulse vortex for 15 s before promptly spin-coating onto a Si wafer. The (100) oriented Si wafers with a thin native SiO_2_ layer were cut to approximately 1.0 cm^2^ squares and plasma-cleaned before coated and spun using a Laurell 650 M series spin-coater at 2000 rpm for 45 s. The coated Si wafers were placed into a vacuum oven for 1 h at 230 °C. Once cooled, the PB was is sonicated in toluene for 10 min in triplicate to ensure all nongrafted polymers were removed from the surface. Then the brushes were isolated and dried via ultrapure nitrogen gas, yielding a polymer brush layer of approximately 4 nm thickness (measured by ellipsometry).

Each polymer brush was analyzed via a Ramé-hart instrument Co. goniometer to obtain contact angles with a 2 μL droplet of direct immersion (DI) water. PB samples were subjected to five separate nonoverlapping droplets to obtain an average CA with a standard deviation for each sample. All polymer brush data is a compilation of at least 2 identical separately prepared samples (10 different CA data points total) (Figure 1a).

**Figure 1:**
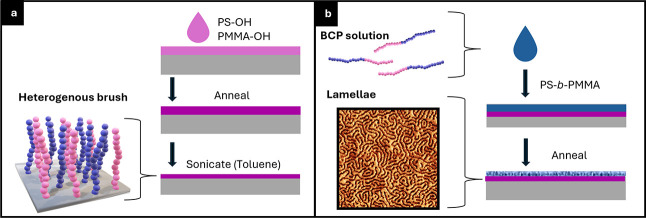
Schematics of the experimental sequence where blue and pink brushes represent many clusters of monomers/sphere. Additionally, PS will be depicted as blue, and PMMA will be depicted as pink throughout this report. Processes are as follows: (a) polymer brush grafting and (b) BCP self-assembly.

#### 2.3.2 BCP Films

Identical polymer brush samples were prepared as previously outlined in Section 2.3.1. Lamellar- and cylinder-forming, BCP (periodicity, *L*_0_ ∼ 30 nm) was spun-coated on the polymer brush and annealed for 30 min in a vacuum oven at 200 and 160 °C, respectively. The annealed BCPs atop the PBs were analyzed via imaging using tapping mode—AFM to observe the self-assembled patterns (Figure 1b).

#### 2.3.3 Solvent Vapor Annealing

Solvent vapor annealing (SVA) was conducted by using a custom-built continuous flow system consisting of two mass flow controllers (Alicat Scientific), a solvent reservoir, and an annealing chamber. Solvent activity was controlled by adjusting the flow ratio of nitrogen and chloroform vapor with total flow maintained at 500 standard cubic centimeters per minute (sccm). Activities ranged from *a* = 0 (pure N_2_) to *a* = 1 (pure chloroform). High-χ BCP swelling was monitored in situ by using a Filmetrics F20 reflectometer. After the film reached equilibrium thickness, a vacuum was applied in the chamber to quench the film back to its original state. Solvent uptake was quantified by the normalized solvent fraction:

2 Φ=(finalthickness−initialthickness)/finalthickness

#### 2.3.4 Direct Solvent Immersion

All PBs prepared for these experiments are prepared according to Section 2.3.1. After the first set of CA is obtained, the same PB sample is submerged in a selective solvent (acetonitrile, MeCN) in sonicate for 10 min and dried via ultrapure nitrogen before CA is obtained. The DI process is repeated with toluene, totaling 3 sets of CA data per PB sample.

## 3 Results and Discussion

PBs were prepared, and their wetting properties were characterized by using water contact angle (WCA) measurements. PS and PMMA showed WCA of 88.6 ± 1.6° and 65.7 ± 7° (Figure 1a), respectively, in agreement with literature (PS ∼ 84.0–88.7° ^19,22,23^ PMMA ∼ 62.8 ± 1.0°^19^). In addition, the reported CA data were statistically indistinguishable between molecular weights for both the PS and PMMA brushes.

Figure 2a displays the WCA of the grafted brush as a function of the homopolymer blend composition. Note that the *x*-axis denotes % PS in the blend before grafting. For brevity, the molecular weight of the blends will be denoted in numbers representing approximate kg/mol in the following sequence PS:PMMA (for exact molecular weights, see Table 1). Generally, PMMA dominates the wetting behavior due to its polar functional groups compared to PS, forming stronger electrostatic interactions with the native silicon oxide.^19,21,24^ Thus, the blends are prepared with higher PS loading (>50%) to introduce competitive PS wetting character alongside PMMA into the brush.

**Figure 2:**
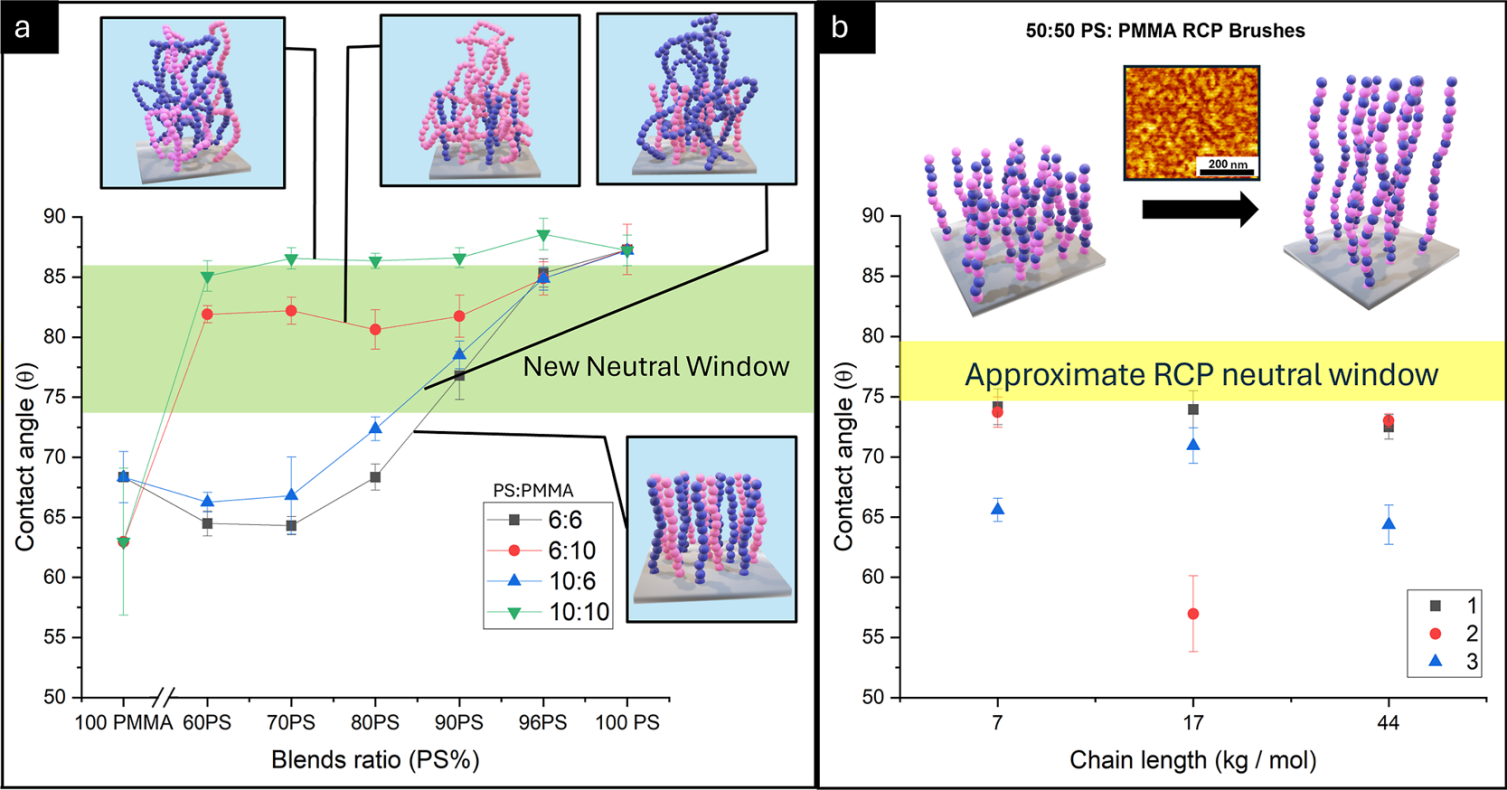
(a) Water contact angle (WCA) of homopolymer blended brushes with congruent and incongruent blended brushes. The *x*-axis denotes % PS in the blend before grafting. (For 60PS-96PS, 10 WCA/identical sample, and 100% cases of PS and PMMA, 15 WCA/identical sample.) (b) WCAs of 50:50 PS-*r*-PMMA with molecular weights of 7 kg/mol, 17 kg/mol, and 44 kg/mol. (15 WCA data points/identical sample.)

Displayed in Figure 2b is a benchmark using RCPs to represent the WCA of a neutral surface. Interestingly, a broad distribution of CAs was observed, highlighting their poor consistency and batch variation. Each color (red, black, and blue) represents an identical sample totaling three sets of each indicated length, demonstrating the degree to which the PBs vary under identical conditions. Additionally, these surface variations are not dependent on the molecular weights of each chain of the RCP. The variations observed here could stem from deviations in the chemical composition between polymer chains, which cannot be controlled during copolymerization. This variation is consistent with recent experimental and simulation studies, such as Song et al., who demonstrated that statistically RCP brushes can still exhibit local compositional fluctuations and dynamic surface responses due to self-organization effects in DSA systems, which can contribute to inconsistent interfacial wetting behavior.^25^ However, we also acknowledge that our experiments, through isolation to the best of our abilities, were not conducted in a cleanroom environment. This may lead to impurities introducing inconsistencies when compared with similar literature on this topic, and hence a vast amount of repeated experiments were conducted to increase certainty. Finally, the inconsistencies could also be attributed to larger PDIs compared to the homopolymers (Table 1).

Interestingly, the 6:6 (black) and the 10:10 (green) congruent brushes (Figure 2a) behaved the most dissimilar among the PB series. The 10:10 brushes retain mostly PS character until ∼60% PS blend with WCA data. Whereas the 6:6 brushes demonstrate a saturated PMMA WCA behavior up to ∼80% PS blend, above which the WCA character begins to develop mixed PS and PMMA character. These effects could stem from the higher degree of conformational freedom in the 10:10 brushes vs the 6:6 brushes, along with the lower grafting density in the 10:10 vs the 6:6 brushes.

Additionally, the air–polymer interface favors PS,^15,26^ whereas PMMA is selective toward the more polar substrate–polymer interface.^27^ Thus, given the expectedly lower graft density of the 10:10 brushes compared with the 6:6 brushes, it is probable that the PMMA chains may have collapsed toward the surface, while the PS chains stretched toward the air–polymer interface; possibly explaining the PS-dominant WCA trends observed in Figure 2a (green) line. The graft densities of PS reported here (0.5–0.6 chains/nm^2^) are consistent with previous reports using extended thermal annealing. For instance, Lee et al. demonstrated grafting densities up to 0.61 chains/nm^2^ for 6k PS brushes using a grafting-to approach with hydroxyl-terminated polystyrenes and 3 day vacuum annealing at 170 °C.^28^ Thus, the 6:6 brushes have less conformational freedom and adopt a more stretched conformation compared with the 10:10 brushes.

In contrast, the incongruent brushes display more complex wetting behavior. For the case of 6:10 brushes (red), the short-chain PS appears to dominate grafting from the WCA data. This is likely due to faster diffusion kinetics, considering that at 6 kg/mol, it is well below its entanglement molecular weight, *M*_e,PS_ ∼ 13 kg/mol.^29^ In this case, the diffusion constant, *D* inversely scales with chain length, *N* by *D* ∼ *N* ^–1^, according to Rouse dynamics.^30^ On the other hand, the long-chain PMMA (10 kg/mol) experiences entangled dynamics (PMMA *M*_e,PMMA_ ∼ 6–9 kg/mol for chains under 150 kg/mol),^29,31^ governed by the de Gennes reptation model, which dictates a larger scaling factor, *D* ∼ *N* ^–2^.^32^ Thus, despite the surface affinity of the long-chain PMMA, it exhibits slower dynamics coupled with fewer hydroxy-terminated groups per unit volume, thus resulting in a lower grafting rate compared to the shorter PS chains in the 6:10 blends.^25^ The preferential grafting of shorter chains is in agreement with recent findings by Chiarcos et al., where detailed analysis of mixed brush compositions were conducted using TOF-SIMS and molecular dynamics.^33^ This supports the findings of the WCA data of 6:10, which maintains high WCA (>80°) despite increasing the PMMA blend ratio up to 40%. However, the grafting of PMMA chains can increase as a result of mechanochemical exchange where PS chains are stretched upon PMMA entering the PS-rich brush layer and undergoes hydrolytic degrafting.^34^

For the 10:6 brushes, the reverse occurs where the short-chain PMMA overwhelms the long-chain PS due to optimal conditions of surface affinity and chain dynamics for PMMA. Here, mixed surface chemistry observable with WCA is only prevalent at high loadings of PS (>80%), where the polymer blend ratio starts competing with the grafting preference of PMMA. The 10:6 blend is comparable to 6:6, where PMMA dominates the WCA trend, albeit with a slightly higher overall WCA, presumably due to the presence of longer PS chains stretching to the air–polymer interface above the PMMA chains to alleviate interfacial energy.

### 3.1 Neutrality of BCP on Homopolymer Blend Grafted Brushes

BCP neutrality investigations were conducted by annealing a lamellar formation of PS-*b*-PMMA, atop a series of brushes to observe the orientation of self-assembly. The resulting BCP thin film was approximately 20 nm thick, as determined by ellipsometry, spectral reflectance, and AFM. The 10:10 brushes were unsuccessful in obtaining vertical orientation from BCP, likely due to the PS dominant surface observed with WCA (Figure 2a). Instead, the 10:10 brush noticeably displayed hole and island features characteristic of horizontally oriented lamellar, which indicates a non-neutral brush. An increase in the relative size of the holes was observed with an increasing blend ratio of PS (Figure S1). The Gibbs free energy of mixing (Δ ***G***_**m**_) (eq S1) was evaluated as a function of blend composition using the Flory–Huggins lattice model assuming χ_PS–PMMA_ = 0.038.^35^ The trends of Δ ***G***_**m**_ are outlined in Figure 3 clearly showing that 10:10 has a positive Δ ***G***_**m**_ for a significant portion of the blend compositions and thus is predicted to undergo macrophase separation, which would lead to patchy surfaces, explaining the hole and island features. Overall, the PS dominant wetting behavior observed in WCA and the substrate affinity of the PMMA likely formed an optimal environment for PMMA to collapse toward the substrate while the PS chains stretched toward the air interface. Entanglement dynamics described in PMMA could have also played a role in reducing its overall contribution on the air surface interface. This is in stark contrast to Ceresoli and Sparnacci et al., who attempted homopolymer blending to form neutral brushes with long-chain PS and PMMA brushes of 16 kg/mol and 15 kg/mol, respectively, using rapid thermal annealing.^21^ Rather than hole-island features throughout all compositions, they reported a narrow window for vertical orientation (>85% PS), albeit mixed with patches resembling hole-island boundaries. The discrepancy could stem from several differences: (i) annealing procedures, where Ceresoli and Sparnacci employed a unique rapid annealing process at temperatures up to 350 °C for a duration ranging from 1 to 600 s, whereas our technique centered around vacuum-thermal annealing at lower temperatures and longer durations;^21^ (ii) Their longer-chain 16:15 blend places both PS and PMMA above their *M*_e_, thus lowering their grafting disparities; and (iii) the high molecular weight BCPs (50 kg/mol-*b*-50 kg/mol PS-*b*-PMMA) used in their work would be more tolerant to local chemical inhomogeneity in the surface due to larger assembled pitch.

**Figure 3:**
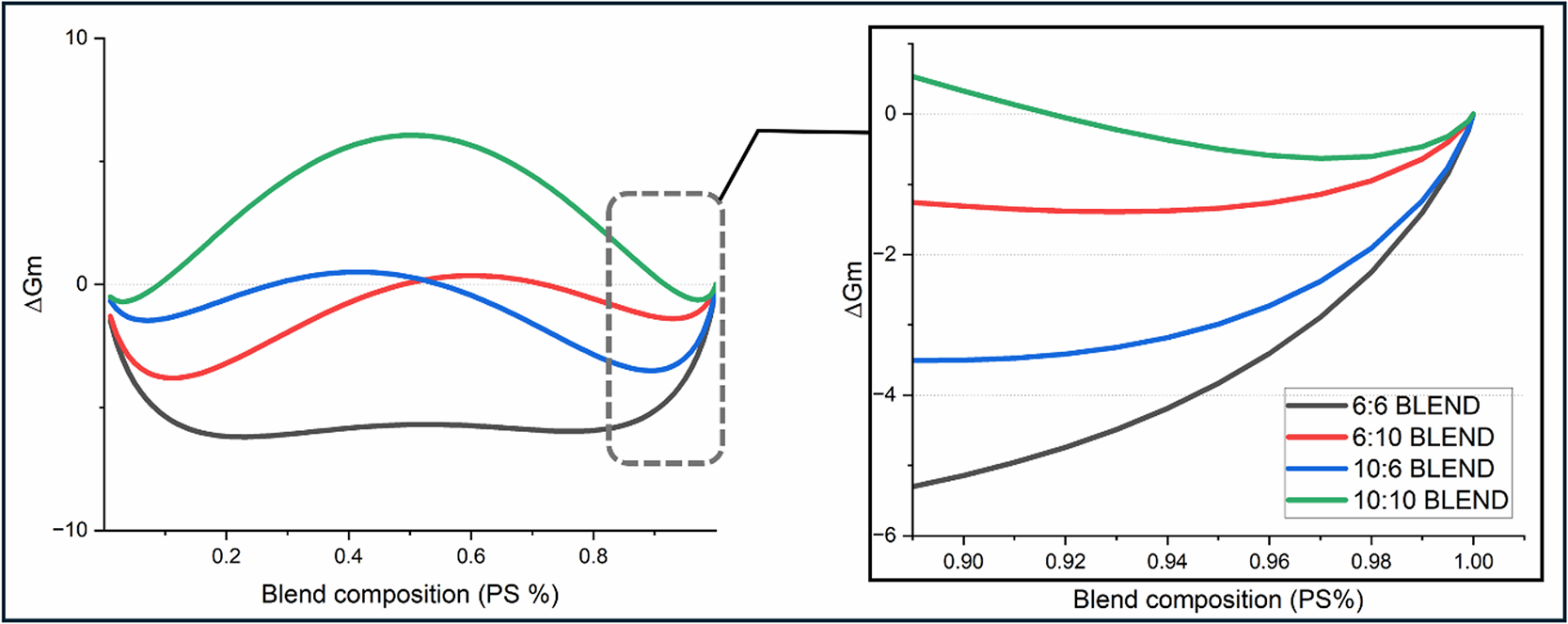
Gibbs free energy (Δ ***G***_**m**_) plotted for congruent and incongruent polymer blends.

#### 3.1.1 Congruent Blends (6:6)

The congruent 6:6 brushes showed a vertical orientation starting above 90% PS (Figure 4a–c). This narrow window is associated with the blend saturation of PS that overcomes the PMMA preference to graft on the substrate. In contrast, the 80% PS brush shows considerable preference toward the PMMA block, in agreement with WCA measurements (Figure 2a). Furthermore, the neutrality window of the 6:6 system is similar to the 16:15 system by Ceresoli and Sparnacci albeit with improved surface coverage.

**Figure 4:**
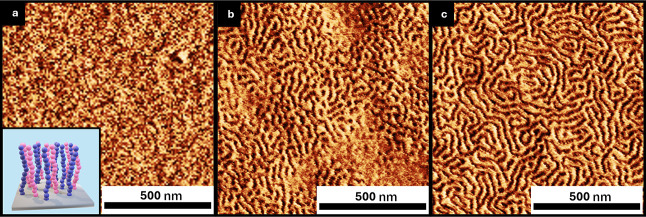
Tapping mode-AFM phase images of PS-*b*-PMMA annealed atop 6:6 congruent polymer brushes with PS blend ratios follows: (a) 80% PS, (b) 90% PS, and (c) 96% PS.

#### 3.1.2 Incongruent Blends (6:10)

The 6:10 incongruent blends formed brushes that demonstrate neutrality in an unprecedented composition window. Figure 5a–f and Figure S2 show vertical orientation from 20% to 96% PS blend, which corresponds up to 99% PS surface concentration (Table 2). At 10% PS blend, the area of neutrality is visibly reduced, but the lamellar structure remains apparent, with distinct island-like features in its vicinity (Figure S2). Furthermore, incongruent blend brushes demonstrate a significantly larger WCA window for BCP neutrality compared to RCP (76–86°, highlighted in green, Figure 2a).^18^ The surface composition of the 6:10 brushes is verified by using XPS (Table 2). The brush compositions were calculated based on integrated C 1s peak areas at 288 eV (C=O component of PMMA) obtained from XPS analysis to quantify the ratio of PMMA to PS on the surface (Figure S3). Although the trend shows PMMA brush composition increasing with lower % PS blend, they remain as the minority component on the surface, down to 50% PS blend. Overall, the composition data indicates that the grafting of PMMA is highly suppressed, in agreement with the chain dynamics analysis in the previous section.

**Figure 5:**
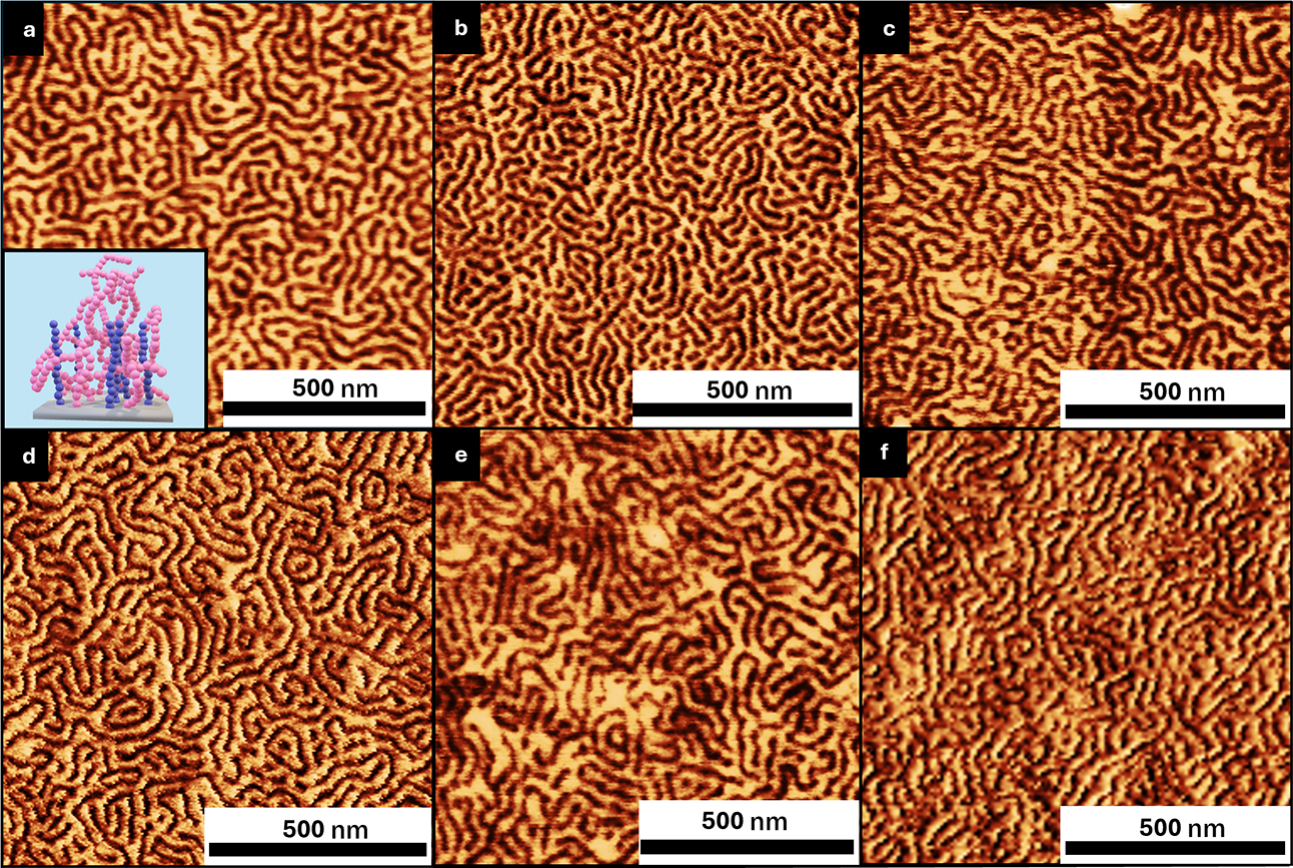
Tapping mode-AFM phase images of PS-*b*-PMMA annealed atop 6:10 incongruent polymer brushes with PS blend ratios as follows: (a) 50% PS, (b) 60% PS, (c) 70% PS, (d) 80% PS, (e) 90% PS, and (f) 96% PS.

**Table 2:** Surface Composition Ratio of 6:10 PS:PMMA Blended Homopolymer Brush Measured Using XPS.

blend type (PS:PMMA)	blend composition	surface composition
6:10	PS 50%-PMMA 50%	PS 81%-PMMA 19%
6:10	PS 60%-PMMA 40%	PS 86%-PMMA 14%
6:10	PS 70%-PMMA 30%	PS 86%-PMMA 14%
6:10	PS 80%-PMMA 20%	PS 90%-PMMA 10%
6:10	PS 90%-PMMA 10%	PS 95%-PMMA 5%
6:10	PS 96%-PMMA 4%	PS 99%-PMMA 1%
3:6	PS 90%-PMMA 10%	PS 81%-PMMA 19%

The neutrality of the 6:10 incongruent brushes was tested on cylinder-forming PS-*b*-PMMA (33k-*b*-17k). Here, vertically oriented cylinder microdomains were observed for 6:10 blend brushes containing 50%, 70%, 80%, and 90% PS, highlighting the width and versatility of the incongruent brush system (Figure 6a–d). Conventionally in RCPs, the composition of the brush needs to be adjusted when switching between lamella and cylinder BCPs due to their dissimilar volume fractions. Thus, the ability of the 6:10 incongruent brush to provide a neutral interface for both lamellar- and cylinder-forming BCP morphologies suggests an extremely robust platform, which will inevitably broaden the process window of DSA patterning.

**Figure 6:**
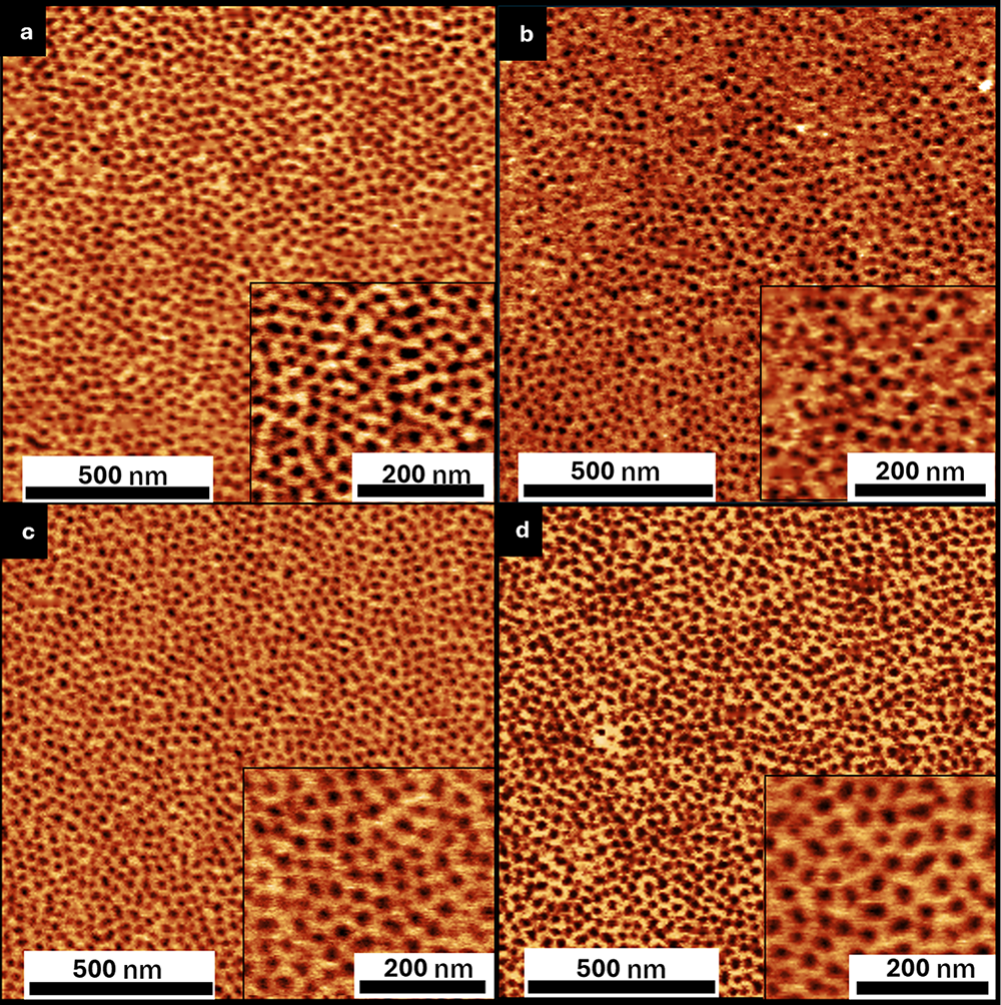
Tapping mode-AFM phase images of PS-*b*-PMMA (33:17) annealed atop 6:10 heterogeneous polymer brushes with PS blend ratios as follows: (a) 50% PS, (b) 70% PS, (c) 80% PS, and (d) 90%.

#### 3.1.3 Incongruent Blends (10:6)

The 10:6 incongruent brushes indicate a highly suppressed PS grafting density due to the less competitive nature of the PS binding over the PMMA and fewer binding sites for PS due to the higher molecular weight (Figure S4). Similarly, the neutrality window aligns closely to the 6:6 brushes where vertical lamellarity was observed above 90% PS loading, as displayed in Figure 7a–d. However, unlike the 6:6 brushes, islands of vertically oriented BCPs can be observed in the 10:6 between 70 and 80% PS loading. This may be due to a sparse PS canopy that neutralizes the BCP in its sparse vicinity.

**Figure 7:**
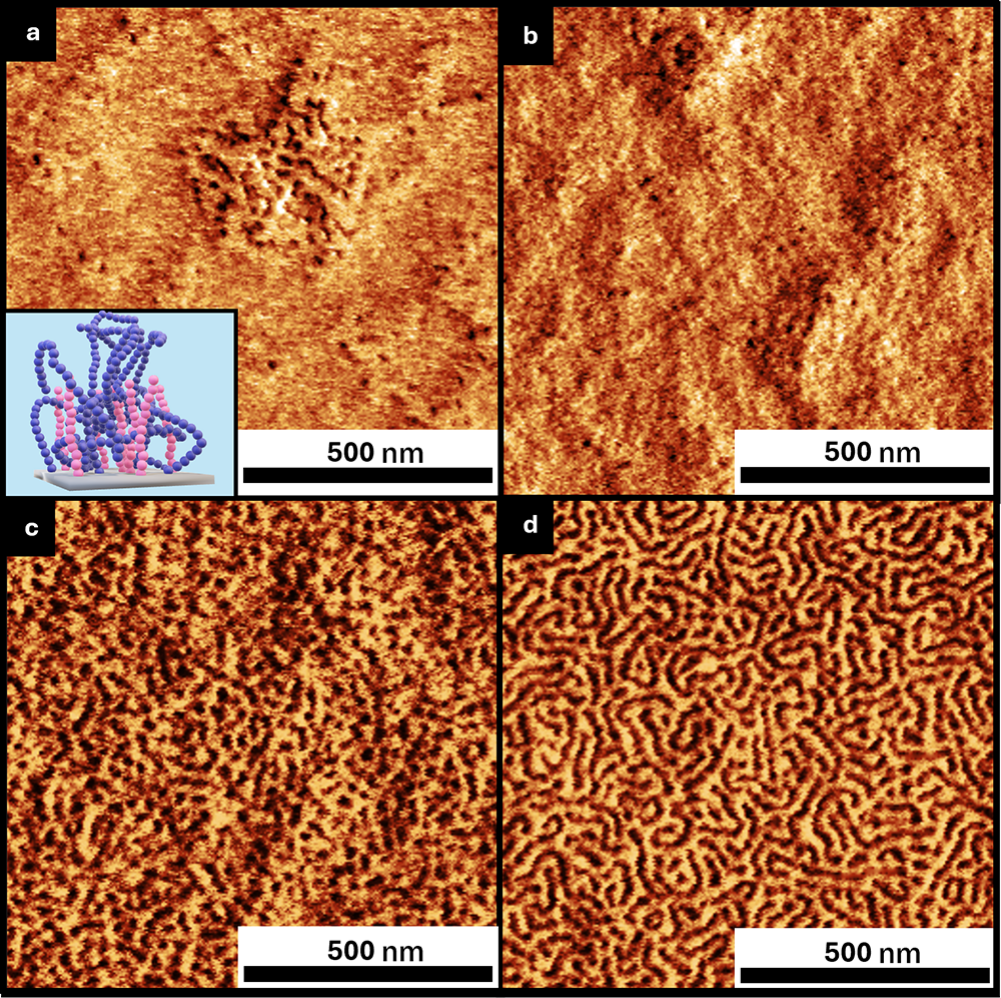
Tapping mode-AFM phase images of PS-*b*-PMMA annealed atop 10:6 incongruent polymer brushes with PS blend ratios as follows: (a) 70% PS, (b) 80% PS, (c) 90% PS, and (d) 96% PS.

### 3.2 Proposed Mechanism

The remarkable neutrality window in the 6:10 system could stem from the reorganization of the long-chain PMMA above the denser short-chain PS, forming a “canopy” structure. Unlike the 10:10, the short-chain PS forms a dense brush structure, essentially forcing the PMMA to the air–polymer interface. Hence, this sparse PMMA brush is conformationally flexible and may be reconfigured to alleviate surface energy mismatch. The sequential insertion of longer chain polymers above a dense brush has been proposed in the DSA of BCPs, where improvements in the chemical and dimension tolerance were observed.^36–38^ Recent simulations on the DSA of di-BCPs using dissipative particle dynamics support that responsive brushes may inevitably widen the neutrality window of substrates.^25^ Similarly, here the sparse and flexible nature of the PMMA chains could minimize interfacial mismatch in the presence of the BCP film by orienting toward the PMMA block, forming essentially a tolerant canopy of long-chain PMMA above the short-chain PS (Figure 8).

**Figure 8:**
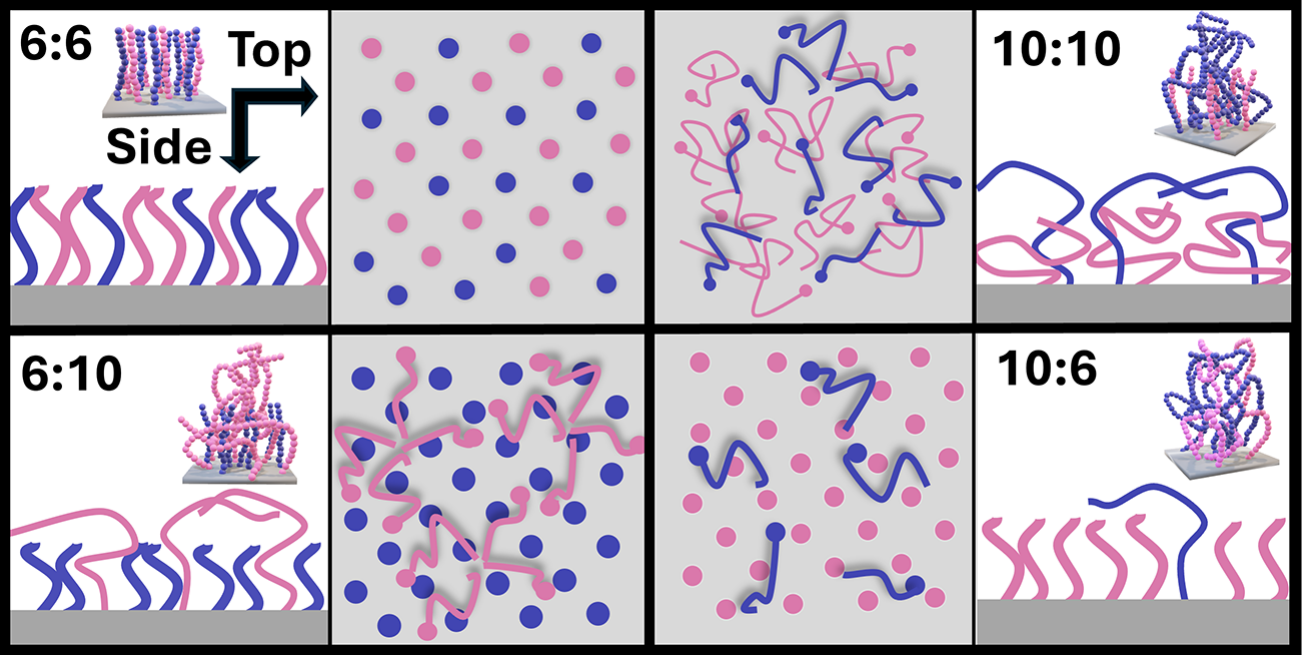
Schematic visual representations of the canopy theory and conformational reorganizations of each brush; a side view (left) and top view (right) are proposed.

#### 3.2.1 Incongruent Blends (3:6)

To verify the responsive nature of the incongruent blend brushes, lower molecular weight blend brushes were prepared while maintaining a similar chain length incongruency (3:6) (Mn_PS_ = 2.7 kg/mol and Mn_PMMA_ = 6 kg/mol). Here, the flexibility of the PMMA canopy is significantly limited by the length of the chains. Ellipsometry measurements show that the 3:6 system has a higher brush thickness compared to the 6:10 system while having a lower molecular weight, confirming the higher graft density of the 3:6 brushes (Table S1). Note that the greater thickness in the 3:6 compared to the 6:10 could also be a result of larger amounts of the longer PMMA chains in the 3:6 system. For example, at 90% PS blend, the 3:6 system has a 19% PMMA surface composition, while the 6:10 system had a 5% PMMA surface composition (Table 2). This is likely due to the 3:6 system governed by Rouse dynamics during grafting, as both PS and PMMA are below their respective *M*_e_, allowing the PMMA surface affinity to play a stronger role. For 50%, 70%, and 90% PS blend in the 3:6 system, the WCA remained below 75° confirming PMMA dominated wetting, supporting the canopy theory, where long-chain PMMA effectively screens the interactions of the shorter dense PS brushes.

Comparing the 3:6 and 6:10 at equal surface composition (90% PS blend in 3:6 and 50% PS blend in 6:10 both display surface compositions of PS 81%-PMMA 19% (Table 2)), however, showed that the former was not neutral toward lamellar BCP, forming hole-island features (Figure S5), whereas the latter successfully produced vertical orientation (Figure 5a). Thus, in addition to incongruency, it can be deduced that the brushes require some degree of conformational freedom to reorganize and minimize interfacial energy with the BCP in support of the proposed mechanism.

### 3.3 Direct Solvent Immersion of PBs

DI experiments were performed to probe the composition of the brush and its reorientation behavior (Figure 9). DI exposes the polymer brush to different chemical environments, changing the long-chain canopy orientation to minimize interfacial mismatch in an attempt to mimic the presence of a polymer film. Assuming reorientation of the canopy is possible, DI allows us to probe the polymer brush behavior based on the different solvent exposures without chemically modifying the binding of the polymer brush to the substrate. During DI, a canopy structure would hypothetically reorient or entrap solvent, thus altering wetting behavior. Here, PMMA will have a selective affinity toward acetonitrile (MeCN), whereas PS has a stronger affinity to toluene. The DI experiments include the as-prepared brushes, which were washed initially in toluene (toluene), followed by exposing the PBs to acetonitrile (DI MeCN), and then toluene again (DI toluene).

**Figure 9:**
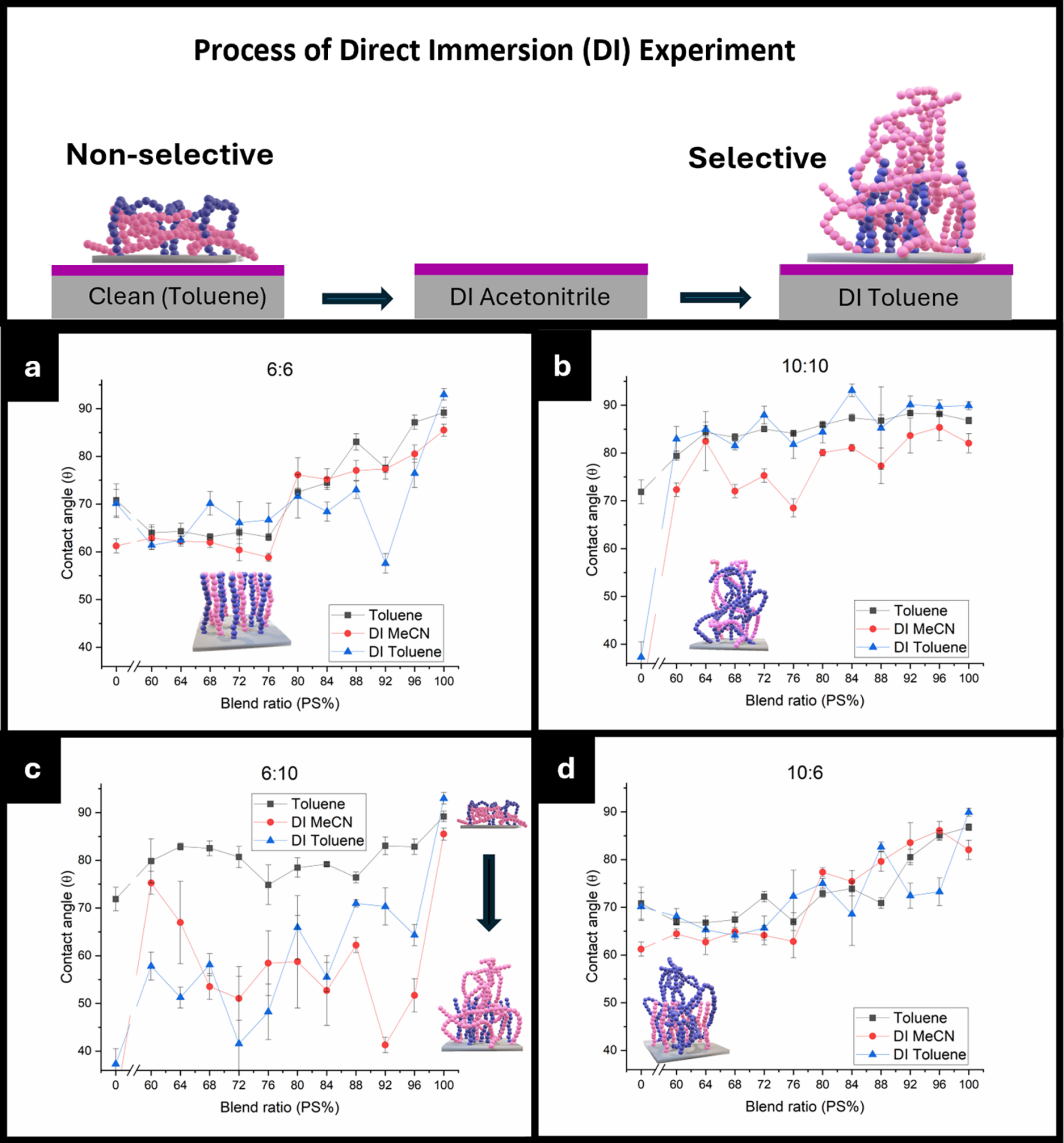
WCA of brushes from each set of homopolymer blends. The as-prepared (black), first DI with acetonitrile (red), and second DI in toluene (blue) WCAs are plotted against the blend ratio. (a) 6:6, (b) 10:10, (c) 6:10, and (d)­10:6.

Figure 9a,d displays the results of 6:6 and 10:6 being highly similar, where no quantifiable alterations in wetting throughout the DI experiments. This is consistent with a short-chain PMMA-dominated surface, where limited MeCN uptake can occur due to a densely grafted brush, which can be visualized in Figure 9. This can also be deduced from the lack of MeCN uptake in the short-chain PMMA (0 PS) and significant shift in the long-chain PMMA (0 PS) after DI MeCN, supporting the idea that the denser short-chain PMMA adopts a stretched conformation, leading to poor swelling. Figure 9d further confirms that long-chain PS brushes are scarce in the 10:6 blends. The wetting behavior remained statistically the same between the DI acetonitrile and DI toluene, suggesting negligible uptake of toluene from long-chain PS brushes. On the contrary, 10:10 displays some decrease in WCA after DI MeCN, followed by a recovery after DI toluene, indicative of a reorganizing brush, albeit limited in this case (Figure 9c). However, the limited MeCN uptake could stem from long-chain PS forming a barrier at the surface or limited long-chain PMMA grafting due to entanglement dynamics, as noted in the previous section.

The 6:10 brushes demonstrate a drastic and systematic decrease in the WCA after DI in MeCN (Figure 9c). However, recovery was not observed after DI toluene. This is attributed to the reorganization and solvent entrapment of MeCN by the long-chain PMMA brushes, which dramatically lowers the WCA of the surface, considering that MeCN has limited solubility in toluene. Recovery back to higher WCA was finally achieved after drying the film in an oven at 100 °C, supporting the notion of solvent absorption in PMMA.

### 3.4 High-χ Polymer Brush

The application of the incongruent brush system was further illustrated on a high-χ BCP (26PS-*b*-26P2VP) using a brush composition of 6 kg/mol PS and 10 kg/mol P2VP, with a blend composition of 96% PS. The BCP films were annealed using acetone vapor at room temperature to facilitate self-assembly.^39^ SVA plasticizes the chains and promotes long-range order, assuming χ is sufficiently large for microphase separation. We utilized a custom-built SVA chamber for controlled solvent vapor flow.^40^ Vertical lamellar morphologies were achieved at two different solvent fractions, ϕ: (a) 0.04 and (b) 0.10 (Figure 10). The vertical orientation of lamellar structure observed in AFM was further confirmed by GISAXS, which showed a primary peak at q* = 0.20 nm^–1^ corresponding to a domain spacing of ∼31.4 nm. At lower ϕ (0.04), the lamellar domains demonstrated a relatively uniform orientation with distinct domain boundaries, signifying effective segregation. Upon increasing ϕ to 0.10, vertical orientation is observed but shows a poorly ordered lamellar due to the BCP approaching its order–disorder transition, in agreement with previous studies.^39^ In the absence of the incongruent brush, the grain size was visibly smaller, and the vertical orientation was not observed for ϕ = 0.04 and 0.10, respectively (Figure S6).

**Figure 10:**
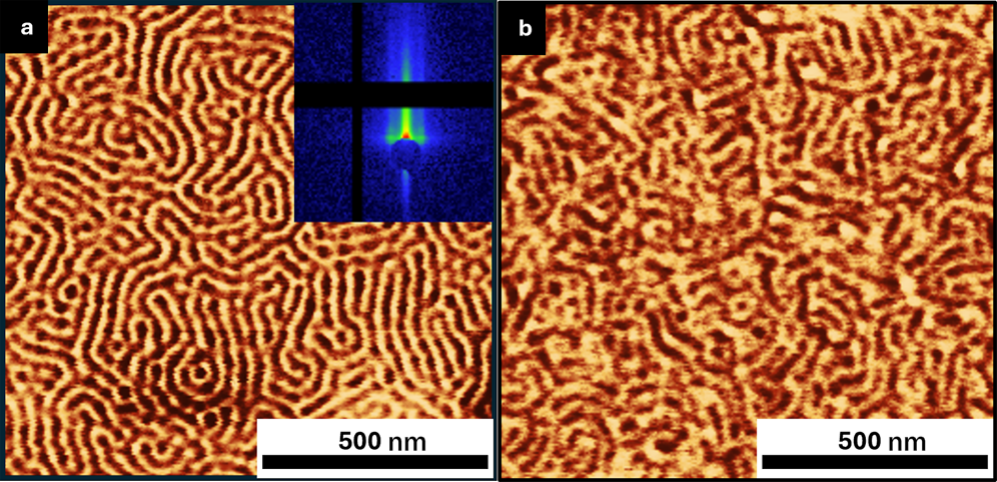
AFM height images of PS-*b*-P2VP films self-assembled on neutral brushes grafted from 6:10 incongruent homopolymer blends (a) 96% PS–P2VP brush prepared and annealed with BCP at solvent fraction, ϕ = 0.04 (GISAXS confirmed the domain periodicity, with a primary scattering peak at q* = 0.20 nm^–1^), and (b) 96% PS–P2VP brush prepared and annealed with BCP at ϕ = 0.1.

## 4 Conclusions

This work demonstrates that incongruent homopolymer blended brushes have an extremely wide neutrality window for effective BCP vertical orientation. The incongruent brushes can be nonpreferential toward both lamellar- and cylinder-forming BCPs without the need of changing blend composition. This method offers a simplistic and tunable approach with an unprecedented range of neutrality. In comparing congruent and incongruent blends of different molecular weights, it can be argued that entanglement dynamics and surface affinity play a significant role in grafting and, hence, the final brush composition. From evaluating the surface neutrality of the brushes through macroscale WCA to nanoscale BCP self-assembly, the expanded neutrality window is rationalized by proposing a canopy theory and its ability to reorient at the surface. The brush canopy and its reorientation were substantiated with lower molecular weight brushes and DI experiments. However, further mechanistic studies of the surface dynamics of the brush are required to validate the canopy theory. These findings contribute to the development and understanding of neutral surfaces for BCP vertical orientation, which has applications in nanomanufacturing, such as semiconductor patterning and photonic devices. Future work includes the development of incongruent brushes for strongly segregating BCPs by alleviating the strict need for matching chemistry.

## Supplementary Material



## Notes

Addition data on AFM micrographs, CA, XPS, and brush thickness (PDF)

## ‡

Biological and Chemical Science and Engineering, Energy and Environment Science and Technology, Idaho National Laboratory

## References

[1] Wang S.-T., Zhang H., Xuan S., Nykypanchuk D., Zhang Y., Freychet G., Ocko B. M., Zuckermann R. N., Todorova N., Gang O. (2022). Compact Peptoid Molecular Brushes
for Nanoparticle Stabilization. J. Am. Chem.
Soc..

[2] Pincus P. (1991). Colloid stabilization
with grafted polyelectrolytes. Macromolecules.

[3] Kreer T. (2016). Polymer-brush
lubrication: a review of recent theoretical advances. Soft Matter.

[4] Peng B., Johannsmann D., Rühe J. (1999). Polymer Brushes with Liquid Crystalline
Side Chains. Macromolecules.

[5] Guo Y., Moffitt M. G. (2007). Semiconductor Quantum Dots with Environmentally Responsive
Mixed Polystyrene/Poly­(methyl methacrylate) Brush Layers. Macromolecules.

[6] Koo K., Ahn H., Kim S.-W., Ryu D. Y., Russell T. P. (2013). Directed self-assembly
of block copolymers in the extreme: guiding microdomains from the
small to the large. Soft Matter.

[7] Darling S. B. (2007). Directing
the self-assembly of block copolymers. Prog.
Polym. Sci..

[8] Liu G., Thomas C. S., Craig G. S. W., Nealey P. F. (2010). Integration of Density
Multiplication in the Formation of Device-Oriented Structures by Directed
Assembly of Block Copolymer–Homopolymer Blends. Adv. Funct. Mater..

[9] Cochran E. W., Garcia-Cervera C. J., Fredrickson G. H. (2006). Stability of the Gyroid Phase in
Diblock Copolymers at Strong Segregation. Macromolecules.

[10] Ruiz R., Kang H., Detcheverry F. A., Dobisz E., Kercher D. S., Albrecht T. R., de Pablo J. J., Nealey P. F. (2008). Density Multiplication
and Improved Lithography by Directed Block Copolymer Assembly. Science.

[11] Liu C.-C., Han E., Onses M. S., Thode C. J., Ji S., Gopalan P., Nealey P. F. (2011). Fabrication of Lithographically Defined Chemically
Patterned Polymer Brushes and Mats. Macromolecules.

[12] Stoykovich M. P., Nealey P. F. (2006). Block copolymers
and conventional lithography. Mater. Today.

[13] Mansky P., Liu Y., Huang E., Russell T. P., Hawker C. (1997). Controlling Polymer-Surface
Interactions with Random Copolymer Brushes. Science.

[14] Lambooy P., Russell T. P., Kellogg G. J., Mayes A. M., Gallagher P. D., Satija S. K. (1994). Observed frustration in confined block copolymers. Phys. Rev. Lett..

[15] Ji S., Liu G., Zheng F., Craig G. S. W., Himpsel F. J., Nealey P. F. (2008). Preparation
of Neutral Wetting Brushes for Block Copolymer Films from Homopolymer
Blends. Adv. Mater..

[16] Ji S., Liao W., Nealey P. F. (2010). Block Cooligomers: A Generalized
Approach to Controlling the Wetting Behavior of Block Copolymer Thin
Films. Macromolecules.

[17] In I., La Y.-H., Park S.-M., Nealey P. F., Gopalan P. (2006). Side-Chain-Grafted
Random Copolymer Brushes as Neutral Surfaces for Controlling the Orientation
of Block Copolymer Microdomains in Thin Films. Langmuir.

[18] Han E., Stuen K. O., La Y.-H., Nealey P. F., Gopalan P. (2008). Effect of
Composition of Substrate-Modifying Random Copolymers on the Orientation
of Symmetric and Asymmetric Diblock Copolymer Domains. Macromolecules.

[19] Liu G., Ji S., Stuen K. O., Craig G. S. W., Nealey P. F., Himpsel F. J. (2009). Modification
of a polystyrene brush layer by insertion of poly­(methyl methacrylate)
molecules. J. Vac. Sci. Technol. B.

[20] Liu G., Thomas C. S., Craig G. S. W., Nealey P. F. (2010). Integration of Density
Multiplication in the Formation of Device-Oriented Structures by Directed
Assembly of Block Copolymer–Homopolymer Blends. Adv. Funct. Mater..

[21] Ceresoli M., Palermo M., Ferrarese Lupi F., Seguini G., Perego M., Zuccheri G., Phadatare S. D., Antonioli D., Gianotti V., Sparnacci K. (2015). Neutral wetting brush
layers for block copolymer thin films using homopolymer blends processed
at high temperatures. Nanotechnology.

[22] Li Y., Pham J. Q., Johnston K. P., Green P. F. (2007). Contact Angle of
Water on Polystyrene Thin Films: Effects of CO2 Environment and Film
Thickness. Langmuir.

[23] Kwok D. Y., Lam C. N. C., Li A., Zhu K., Wu R., Neumann A. W. (1998). Low-rate dynamic contact angles on polystyrene and
the determination of solid surface tensions. Polym. Eng. Sci..

[24] Mittal, K. L. Adhesion Aspects of Thin Film; v.2: Volume 1; VSP, 2001.

[25] Song Q., Zhou J., Dong Q., Tian S., Chen Y., Ji S., Xiong S., Li W. (2024). Directed Self-Assembly
by Sparsely
Prepatterned Substrates with Self-Responsive Polymer Brushes. Langmuir.

[26] Wu S. (1970). Surface and
interfacial tensions of polymer melts. II. Poly­(methyl methacrylate),
poly­(n-butyl methacrylate), and polystyrene. J. Phys. Chem..

[27] Ahn D. U., Wang Z., Campbell I. P., Stoykovich M. P., Ding Y. (2012). Morphological evolution of thin PS/PMMA films: Effects of surface
energy and blend composition. Polymer.

[28] Lee H., Lee W., Soo Han Y., Kim E., Ryu D. Y. (2016). Autophobic dewetting
of polystyrenes on the substrates grafted with chemically identical
polymers. Polym. J..

[29] Wool R. P. (1993). Polymer
entanglements. Macromolecules.

[30] Rouse P. E. (1953). A Theory of the
Linear Viscoelastic Properties of Dilute
Solutions of Coiling Polymers. J. Chem. Phys..

[31] Masuda T., Kitagawa K., Onogi S. (1970). Viscoelastic
Properties of Poly­(methyl
methacrylates) Prepared by Anionic Polymerization. Polym. J..

[32] de
Gennes P. G. (1971). Reptation of a Polymer Chain in the Presence of Fixed
Obstacles. J. Chem. Phys..

[33] Chiarcos R., Antonioli D., Baldanza A., Brondi C., Munaò G., Milano G., Laus M., Perego M. (2025). Thermodynamic vs Kinetic
Control of Brush Composition in Grafting to Reactions of Disperse
Polymer Systems in Melt. Macromolecules.

[34] Laus M., Chiarcos R., Gianotti V., Antonioli D., Sparnacci K., Munaò G., Milano G., De Nicola A., Perego M. (2021). Evidence of Mechanochemical
Control in “Grafting
to” Reactions of Hydroxy-Terminated Statistical Copolymers. Macromolecules.

[35] Zemła J., Lekka M., Raczkowska J., Bernasik A., Rysz J., Budkowski A. (2009). Selective
Protein Adsorption on Polymer Patterns Formed
by Self-Organization and Soft Lithography. Biomacromolecules.

[36] Ji S., Liu C.-C., Liu G., Nealey P. F. (2010). Molecular Transfer
Printing Using Block Copolymers. ACS Nano.

[37] Wan L., Ruiz R., Gao H., Albrecht T. R. (2017). Self-Registered
Self-Assembly of Block Copolymers. ACS Nano.

[38] Chang B. S., Loo W. S., Yu B., Dhuey S., Wan L., Nealey P. F., Ruiz R. (2023). Sequential
Brush Grafting for Chemically
and Dimensionally Tolerant Directed Self-Assembly of Block Copolymers. ACS Appl. Mater. Interfaces.

[39] Xiong S., Wan L., Ishida Y., Chapuis Y.-A., Craig G. S. W., Ruiz R., Nealey P. F. (2016). Directed Self-Assembly of Triblock Copolymer on Chemical
Patterns for Sub-10-nm Nanofabrication via Solvent Annealing. ACS Nano.

[40] Hendeniya N., Chittick C., Hillery K., Abtahi S., Mosher C., Chang B. (2024). Revealing the Kinetic Phase Behavior of Block Copolymer Complexes
Using Solvent Vapor Absorption-Desorption Isotherms. ACS Appl. Mater. Interfaces.

