# Supplementary material for: Wide Neutrality Window for Block Copolymer Vertical Orientation Using Incongruent Homopolymer Blended Brushes
Source: ACS Appl Mater Interfaces. 2025 Sep 9;17(39):55328–37. doi: 10.1021/acsami.5c11923 (PMC12492326; doi:10.1021/acsami.5c11923)
Supplement: Supplementary file 1 [file am5c11923_si_001.pdf]

# Supporting Information

## Wide Neutrality Window for Block Copolymer Vertical Orientation using Incongruent Homopolymer Blended Brushes

*Kaitlyn Hillery,[§] Sharif Tasnim Mahmud,[§] Nayanathara Hendeniya, Ava Huth, Caden Chittick, Shaghayegh Abtahi, Boyce S. Chang**

Department of Materials Science and Engineering, Iowa State University, Ames, Iowa, IA 50011 United States

[§]K.H and S.T.M. contributed equally to this paper.

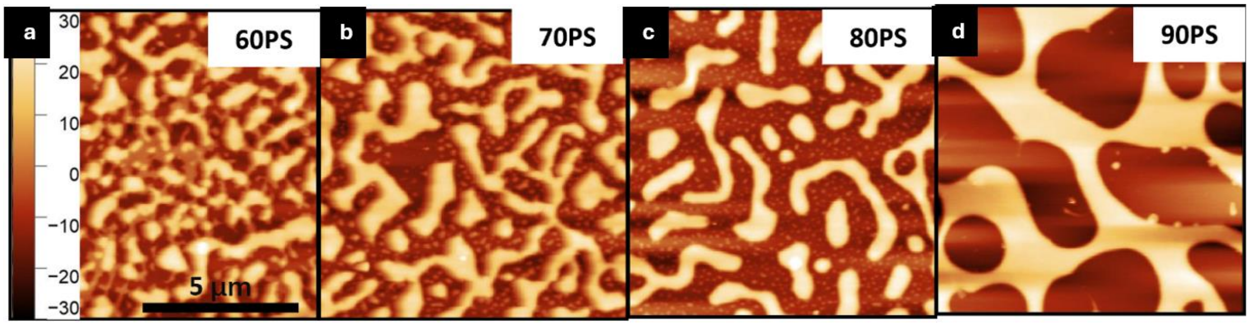


**Figure S1:** Tapping mode-AFM Long-chain macro-phase separation images of PS-*b*-PMMA annealed atop 10:10 heterogeneous polymer brushes with PS blend ratios from 60 – 90 PS blend compositions (a-d).

$$\Delta \boldsymbol{G_m} = \Delta \boldsymbol{H_m} - \boldsymbol{T}\Delta \boldsymbol{S_m} \text{ where } \Delta \boldsymbol{H_m^{interaction}} = \boldsymbol{RTn_1}\, \boldsymbol{x_1} \emptyset_2 \chi_{ij}, \qquad \text{(Equation S1)}$$

$$\Delta \boldsymbol{S_m^{conf}} = -\boldsymbol{R}(\boldsymbol{n_1}\, \boldsymbol{ln}(\emptyset_1) + \boldsymbol{n_2}\, \boldsymbol{ln}(\emptyset_2)) \qquad \text{(Equation S2)}$$

$$\chi_{ij} \cong \frac{\boldsymbol{V_1}}{\boldsymbol{RT}}(\delta_1 - \delta_2)^2 \qquad \text{(Equation S3)}$$

**Equation S1:** Gibbs free energy of mixing for polymer blends, including the enthalpy of mixing for PS and PMMA blends and the entropy of mixing derived from Flory-Huggins theory for polymer-polymer systems.
**Equation S2**: Configurational entropy from the lattice model.
**Equation S3**: Effective Flory-Huggins from the Hildebrand solubility parameters.

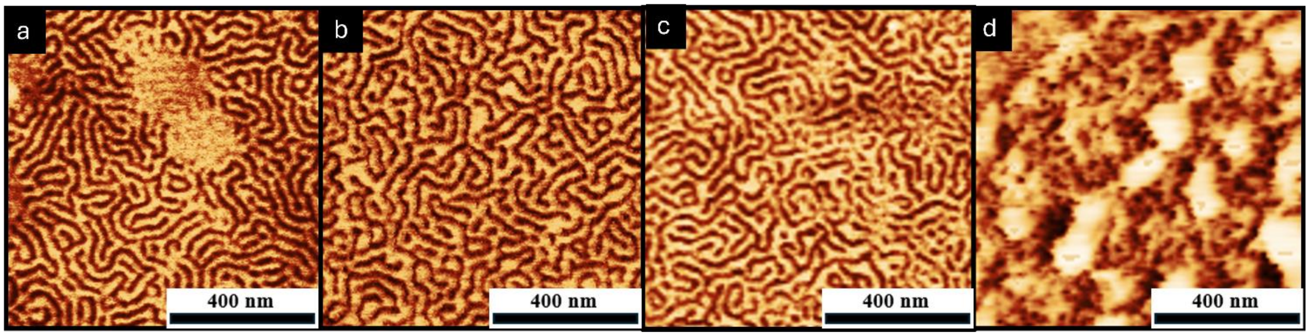


**Figure S2:** Tapping mode-AFM phase images of PS-*b*-PMMA annealed atop 6:10 heterogeneous polymer brushes with PS blend ratios as the following: a) 40% PS, b) 30% PS, c) 20% PS, d) 10% PS.

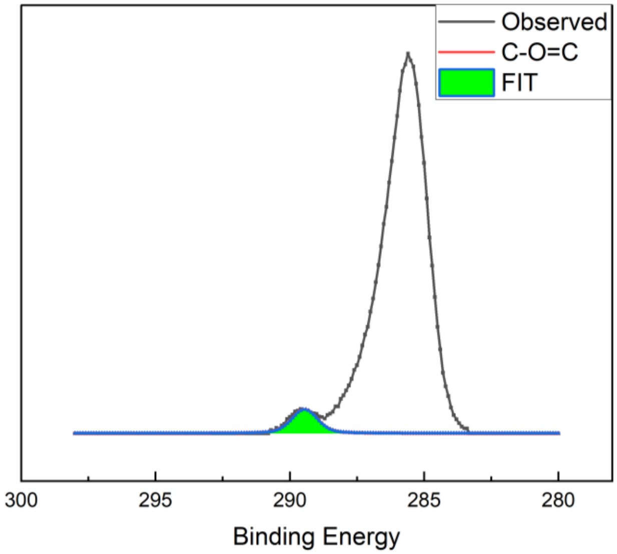


**Figure S3:** Shows the XPS spectrum for the 50% PS in the 6:10 system. The peak at 288 eV, green-shaded region represents the fitted peak for the C=O component of PMMA. The remaining PMMA 4 carbon signals are convoluted with the PS carbons, which makes up the broad peak centered at 285.4 eV. Here, the C=O peak is taken as 1/5 of the total PMMA carbons, which allows deconvolution of the PMMA/PS ratio. The observed (black) and fitted (green) curves align closely, confirming effective separation of the C=O signal. We also performed XPS on 100% PMMA, which validates that the peak 288 eV represents 1/5 of the total integration.

**Table S1:** Difference between film thickness 6:10 system and 3:6 system polymer brushes.

| **Brush (6:10) PS:PMMA** | **Film thickness (nm)** | **Brush (3:6) PS:PMMA** | **Film thickness (nm)** |
|---|---|---|---|
| 50k PS | 4.7 ±.01 | 50k PS | 5.4 ±.02 |
| 70k PS | 4.3 ±.01 | 70k PS | 5.3 ±.02 |
| 90k PS | 4.4 ±.01 | 90k PS | 4.4 ±.05 |

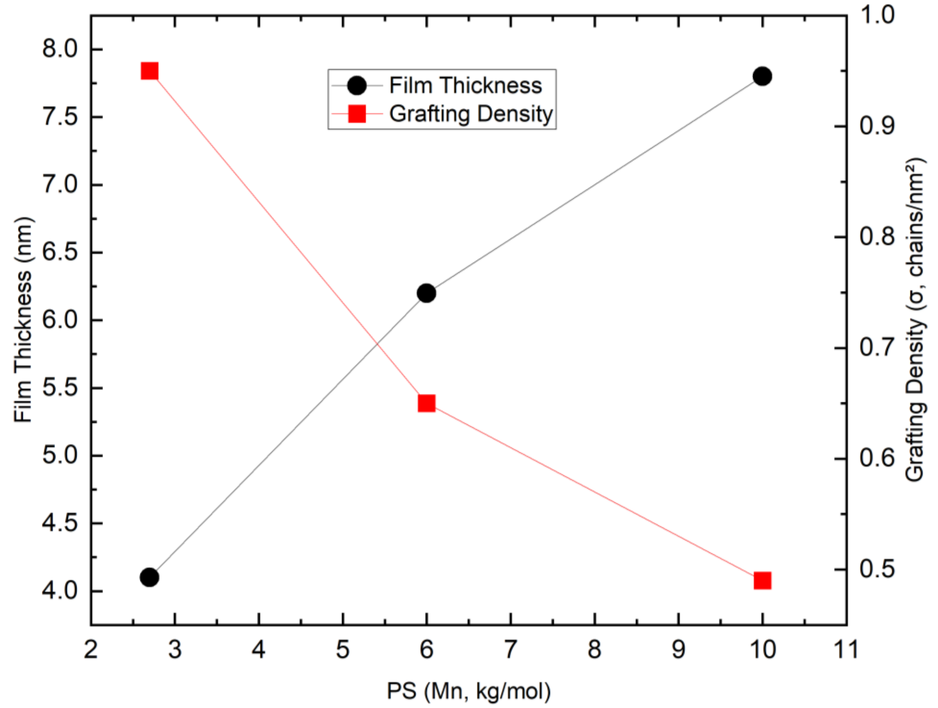


**Figure S4:** Relationship Between Film Thickness and Grafting Density of Polystyrene (PS) Brushes as a Function of Molecular Weight (Mn).

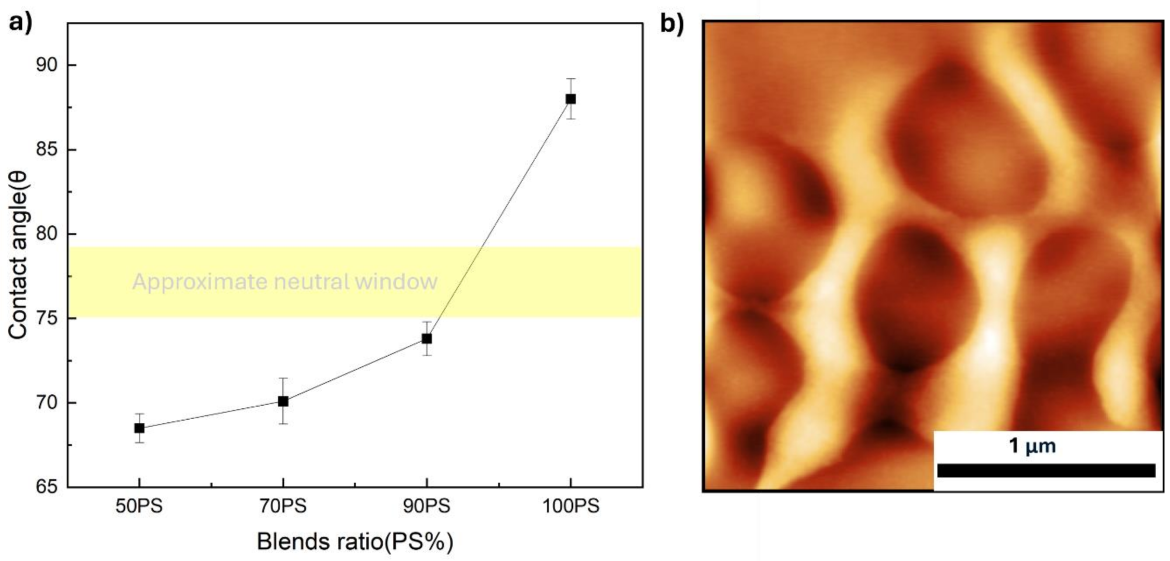


**Figure S5:** a) Water contact angles (WCA) of PS-PMMA blends with PS chain lengths of 2.7 kg/mol, PMMA chain length 6 kg/mol. b) Tapping mode-AFM phase images of PS-*b*-PMMA annealed atop 3:6 heterogeneous polymer brushes with 90% PS blend ratio.

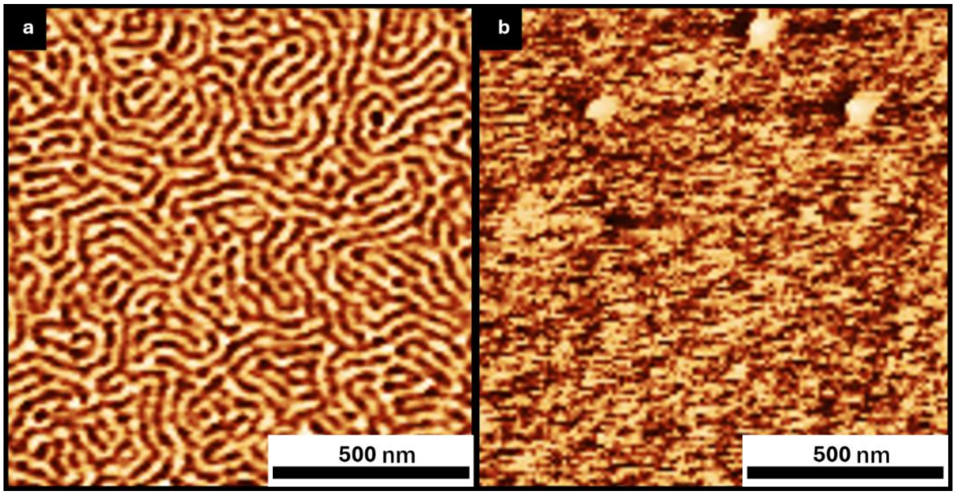


**Figure S6:** AFM height images of PS-*b*-P2VP films self-assembled on Si-wafer a) annealed with BCP at $\phi$ = .04, b) annealed with BCP at at $\phi$ = 0.1.
